# Acute kidney injury over the past decade: from definition evolution to pathogenesis insights and innovative therapeutic strategies

**DOI:** 10.1007/s00018-026-06104-5

**Published:** 2026-03-06

**Authors:** Jinwen Chen, Xuan Xu, Yifang Cai, Qing Zhang, Xudong Wang, Dongshan Zhang

**Affiliations:** 1 Department of Emergency Medicine, Hunan Aerospace Hospital, Hunan Normal University, Changsha, 410205 Hunan China; 2 Department of Critical Medicine, The Second Xiangya Hospital, Central South University, Changsha, 410011 Hunan China; 3 Furong Laboratory, Changsha, 410011 Hunan China; 4 Fujian Medical University, Fuzhou, 350122 Fujian China; 5 Department of Dermatology, The Second Xiangya Hospital, Central South University, Changsha, 410011 Hunan China

**Keywords:** AKI, RCD, Imflammation, DNA damage

## Abstract

Acute kidney injury (AKI) is a major global health concern affecting approximately 13.3 million individuals annually and contributing to 1.7 million deaths, with disproportionately high incidence in low- and middle-income countries, children, and critically ill patients. Its complex pathogenesis centers on regulated cell death(RCD) (apoptosis, pyroptosis, necroptosis, ferroptosis, cuproptosis), inflammation, DNA damage, and metabolic disorders, and recent advances in single-cell sequencing have uncovered key molecular pathways and therapeutic targets. Early diagnosis is pivotal for preventing progression to chronic kidney disease (CKD), yet delays remain a major barrier to improved outcomes. The evolving definition—from acute renal failure (ARF) to AKI and the recent proposal of acute kidney disease (AKD)—has enhanced identification of renal dysfunction, facilitating timely intervention. AKI is classified into prerenal, intrinsic renal, and postrenal types, with subdivisions including sepsis-induced and drug-induced variants. Innovative diagnostic tools, such as urinary biomarkers (TIMP-2, IGFBP7, NGAL, KIM-1), non-coding RNAs, advanced imaging, and AI-driven models, offer promise for early detection, though standardization and biomarker validation challenges persist. Current treatments are largely supportive, but emerging therapies—including mesenchymal stem cell therapies, traditional Chinese medicine, anti-inflammatory agents, and ferroptosis inhibitors—show potential for renal repair. Future directions emphasize the Acute Disease Quality Initiative’s digital AKI management model, integrating AI-enabled dynamic monitoring and multilevel, integrated care to optimize volume management and improve patient outcomes.

## Introduction

Acute kidney injury (AKI) is an essential public health threat on a worldwide scale. Every year, it affects roughly 13.3 million people globally and is thought to be a contributing factor in 1.7 million deaths. Based on the KDIGO-equivalent definition in 154 studies (*n* = 3,585,911), the pooled incidence rates of AKI are 21.6% in adults and 33.7% in children during hospital care, with corresponding AKI-associated mortality rates of 23.9% in adults and 13.8% in children, showing a declining trend over time and an inverse relationship with national income and health expenditure proportion [1]. Among critically ill patients, incidence rate may reach up to 43.7% [2, 3]. Notably, AKI remains disproportionately prevalent in low- and middle-income countries, where it is often accompanied by short-term high mortality, increased complication rates, and sharply rising healthcare costs [4].

The pathogenesis of AKI is complex and multifactorial, involving inflammation, multiple cell death pathways, and DNA damage. In recent years, intensified research efforts have led to a more comprehensive understanding of AKI, offering valuable insights into potential therapeutic targets. Concurrently, the advent of advanced technologies, such as single-cell sequencing, has provided powerful tools for elucidating molecular mechanisms, particularly those governing interactions between renal resident cells and immune cells, as well as for identifying key injured cell types and associated molecular pathways.

Early diagnosis is critical, as it enables timely intervention, prevents progression to chronic kidney disease (CKD), and reduces mortality [5]. However, delayed diagnosis and treatment remain major contributors to AKI-related deaths [6]. Encouragingly, the past decade has seen the emergence of novel biomarkers, advanced imaging techniques, molecular probes, and artificial intelligence (AI)-driven tools that offer promise for early detection of AKI. Despite these advances, challenges persist in validating early biomarkers and predictive systems, as well as in standardizing AKI definitions and enhancing mechanistic insights.

This review systematically summarizes key advances in acute kidney injury (AKI) research over the past decade, with emphasis on definition shifts from acute renal failure (ARF) to AKI/AKD, refined pathogenic mechanisms including regulated cell death and inflammation, and innovative diagnostic/therapeutic strategies. By integrating these multi—dimensional progresses, the work provides clinicians and researchers a comprehensive update, while highlighting unresolved issues and future directions centered on AI—driven management and targeted therapies to enhance AKI outcomes.

## Definition and diagnosis: from ARF to AKI to AKD

As diagnostic criteria have been advanced and refined by the medical and scientific communities, the condition previously termed acute renal failure (ARF) was redefined as AKI by the Kidney Disease: Improving Global Outcomes (KDIGO) organization in 2012. This reclassification was based on specific criteria, including absolute or relative changes in serum creatinine levels (≥ 0.3 mg/dL or ≥ 50% increase within 48 h) or a reduction in urine output (< 0.5 mL/kg/h for six consecutive hours). However, it has since been recognized that these criteria do not fully capture [1] all patients with functional and/or structural renal abnormalities. To address this limitation, KDIGO convened a consensus conference in June 2019 to propose expanded definitions and classifications for acute kidney disease (AKD) and related disorders, in addition to AKI [7]. Further efforts to standardize the conceptual framework of AKD led to a consensus workshop in 2021, where AKD was formally defined as abnormalities in renal function and/or structure persisting for three months or less [8]. This broader classification encompasses not only AKI but also individuals with new-onset kidney disease (NKD) who exhibit renal dysfunction without meeting the diagnostic criteria for AKI, as well as those with structural or functional abnormalities persisting beyond seven days. The introduction of AKD as a diagnostic category has important implications for both research and clinical practice. It facilitates earlier intervention and treatment of kidney disorders, reduces the risk of AKD progression to CKD, and ultimately enhances renal recovery, reduces long-term complications, and improves quality of life. While AKD extends diagnostic coverage to early-stage and persistent renal abnormalities, this review focuses on AKI due to its acute clinical impact and extensive research progress over the past decade.

## Pathogenesis

As scientific research continues to advance, AKI is increasingly recognized as a multifaceted syndrome with diverse etiologies and complex pathophysiological mechanisms. AKI is systematically classified into three primary types: prerenal, intrinsic renal, and postrenal AKI. Furthermore, based on specific causative factors, AKI can be subdivided into sepsis-induced, ischemia–reperfusion (IR), drug-induced, surgery-related, and other variants. Over the past decade, extensive investigations have significantly deepened our understanding of the pathogenesis of AKI. These efforts have yielded valuable insights into the molecular and cellular mechanisms underlying renal injury, paving the way for novel therapeutic targets and the development of improved clinical management strategies.

### Regulated cell death in AKI

AKI involves multiple regulated cell death forms, mainly including apoptosis mediated by mitochondrial, death receptor, and ER stress pathways, as well as pyroptosis, necroptosis, ferroptosis, and cuproptosis, each with distinct molecular mechanisms contributing to kidney injury. These RCD processes are regulated by epigenetic modifications, non-coding RNAs, and specific signaling pathways, highlighting the complexity of AKI pathogenesis (Fig. 3).

#### Apoptosis

Apoptosis is a classical form of programmed cell death implicated in AKI, involving three major pathways: mitochondrial-mediated, death receptor-mediated, and endoplasmic reticulum (ER) stress-induced apoptosis. Recent studies have revealed that the mode of cell death varies depending on the etiology of AKI.

A recent study has revealed that the impact exerted by alcohol on AKI is associated with the process of apoptosis. To comprehensively evaluate the effects of alcohol on AKI, this study employed a 10-day chronic binge alcohol model (Lieber-DeCarli diet combined with 31.5% ethanol via gavage), followed by IR injury (IRI). The underlying mechanism is attributable to the higher levels of cytosolic alcohol dehydrogenase(ADH) metabolic products in females, which activate signaling pathways via the integrin β1 and the downstream JNK signaling pathway. In integrin β1—knockdown mice, there was a decrease in JNK phosphorylation and a reduction in AKI severity. Conversely, activation of this pathway aggravated renal injury and tubular apoptosis in both in vivo and in vitro models [9].

Epigenetically, the C-terminus of Hsc70-interacting protein(CHIP) functions as an E3 ubiquitin ligase, catalyzing the proteasomal degradation of NUR77, thereby protecting against BCL—2—related anti—apoptotic processes [5]. The interaction of the small GTP-binding protein GDP dissociation stimulator with PERK-dependent ER stress also influences cisplatin-induced AKI progression. WW domain-containing E3 ubiquitin protein ligase 2 (WWP2) promotes the ubiquitination and degradation of the autophagy inhibitor CDC20, thereby suppressing apoptosis during AKI progression [10, 11]. While, N-acetylgalactosaminyltransferase-3 suppressed AKI progression by promoting O-glycosylation of the epidermal growth factor receptor (EGFR), although EGFR activation negated this protective effect [12]. Non-coding RNAs have emerged as pivotal regulators in AKI, functioning both as diagnostic biomarkers and modulators of apoptotic pathways [13–16].

Recent research has unveiled novel mechanisms implicated in apoptosis in the context of AKI involving traditional molecules. In 2023, a novel mechanism involving the traditional AKI biomarker KIM-1 (Kidney Injury Molecule-1) was elucidated. Yin Yang 1 binding negatively regulates KIM-1 transcription, while KIM-1 itself binds to the extracellular domain of death receptor 5, inducing apoptosis [17]. Beyond the canonical mitochondrial pro-apoptotic molecule Bax, Voltage-Dependent Anion Channel 1 (VDAC1) has been implicated in AKI. Its interaction with DsbA-L promotes apoptosis in proximal tubular cells, exacerbating injury [18]. Matrix metalloproteinase-7 (MMP-7), upregulated in AKI, inhibits apoptosis by degrading FasL and ameliorates typical death receptor-mediated cell death [19].

The apelinergic system is essential for the regulation of blood pressure and maintenance of water homeostasis. It comprises two indispensable bioactive peptides, apelin and elabela, along with a specific receptor. Notably, apelin-13 has garnered significant attention in renal research for its protective effects in AKI, by modulating inflammation, apoptosis, DNA damage, and related signaling pathways [20].

#### Autophagy

Autophagy plays a complex and multifaceted role in the onset and progression of AKI, with its mechanisms of action and impacts varying depending on cell type and injury model.

Autophagy activation protects proximal tubular epithelial cells(PTECs) from damage caused by various AKI etiological factors, such as IRI and cisplatin nephrotoxicity. Specifically, vacuolar membrane protein 1 (VMP1) exerts a crucial protective effect on PTECs under AKI conditions by maintaining high autophagic flux [21, 22]. Additionally, circ-ZNF609, which is highly expressed in the kidneys following IRI, participates in the pathological process of AKI by encoding ZNF609-250aa protein and mediating autophagy through the AKT3/mTOR signaling pathway [23].

In macrophages, autophagy serves as an adaptive response in AKI, and its deficiency leads to elevated serum creatinine levels and aggravated renal injury. In LPS- and IR-induced AKI, autophagy promotes the ubiquitination and degradation of TARM1 in macrophages via MARCHF1 and MARCHF8, thereby inhibiting TARM1-mediated renal inflammatory injury [24]. Macrophage-tubular epithelial cell(TEC) interactions also influence AKI progression. In ATG7-knockdown mice, autophagy-deficient macrophages exacerbate AKI by releasing exosomal miR-195a-5p, which subsequently activates the miR-195a-5p/SIRT3 signaling pathway in tubular epithelial cells(TECs) [25].

#### Pyroptosis

Pyroptosis, a proinflammatory form of regulated cell death (RCD), is typically mediated by the NLRP3 inflammasome, which activates caspase-1 and gasdermin D (GSDMD). Activated GSDMD forms membrane pores, leading to cell lysis and the release of proinflammatory cytokines IL-1β and IL-18. During IRI—AKI, the incidence of tubular cells undergoing apoptosis, pyroptosis, ferroptosis, and necroptosis varies narrowly, ranging from 12.01% to 14.25% [26]. Among them, pyroptosis is the leading cause of RCD during IRI [26].

New forms of GSDMD activation and the formation of GSDM pore have been explored in AKI. In addition to caspase-1, caspase-4/5/11 contributes to GSDMD cleavage and pyroptosis in contrast-induced AKI [27]. While in sepsis-associated AKI, inhibition of caspase-1 or silencing of caspase-11 suppress pyroptosis and improve renal outcomes [28, 29]. Intestingly, during IRI, ER stress activates the transcription factor CHOP, which upregulates caspase 3, leading to the cleavage of GSDME into GSDME-N and subsequent pyroptosis [30]. Dual-specificity phosphatase 2 (DUSP2) functions as a nuclear dephosphorylating enzyme. Mechanistically, DUSP2 dephosphorylates and inactivates STAT1, thereby attenuating STAT1-mediated transcriptional activation of GSDMD and suppressing subsequent pyroptotic cell death [31].

Mitochondrial reactive oxygen species (mtROS) overload promotes the activation of the NLRP3 inflammasome. RNA sequencing of wild-type (WT) and FAM3A conditional knockout (FAM3A^CKO^) mice affected by IRI revealed that FAM3A deletion exacerbates AKI by by impairing tubular cell responses to mtROS. Furthermore, the PI3K/AKT/NRF2 antioxidant signaling pathway is involved in the process of FAM3A—mediated inhibition of pyroptosis and NLRP3 inflammasome formation [26]. While in sepsis-induced AKI, RNA sequencing has identified acetyl-CoA synthetase 2(ACSS2) participates in inflammasome formation via KLF5/NF-κB signaling [32].

#### Necroptosis

Phosphorylation of RIPK1, RIPK3, and MLKL is central to the canonical necroptotic pathway. This process leads to MLKL oligomerization and the formation of membrane pores, resulting in the leakage of damage-associated molecular patterns (DAMPs) and cellular electrolytes, culminating in necroptotic cell death. The gastrin-releasing peptide receptor (GRPR), a novel receptor implicated in AKI, is activated and cooperates with Toll-like receptor 4 (TLR4). This interaction enhances the expression of STAT1 and its phosphorylated form (p-STAT1), which in turn upregulates the transcription of MLKL (a key mediator of necroptosis), the macrophage recruitment factor CCL2, and gastrin-releasing peptide (GRP). GRP further amplifies GRPR/STAT1 signaling, contributing to inflammation and necroptosis in AKI [33]. TEA domain family member 1 (TEAD1) is highly expressed in cisplatin-induced AKI, and its deletion results in the phosphorylation of RIPK1, RIPK3, and MLKL,thus induce the pyroptosis [34]. However, the underlying phosphorylation mechanism of those necroptosis—related molecules has not been fully elucidated in this study. Meanwhile, TGF-βRII/Smad2 aggravates necroptosis by promoting the transcription of RIPK1, RIPK3, and MLKL following cisplatin treatment [35].

#### Ferroptosis

Ferroptosis and cuproptosis are metal-dependent RCD processes. Iron, an essential trace element, plays a central role in ferroptosis, a process formally named by Dixon et al. in 2012 as excessive lipid peroxidation-mediated iron-dependent cell death. Recent studies have established that ferroptosis contributes significantly to the development of various pathological conditions [36] and is identified by iron overload, ROS build-up, and lipid peroxidation, with GPX4 being the most important enzyme capable of degrading lipid peroxides.

The antioxidant function of GPX4 is modulated via epigenetic mechanisms. Mitochondrial acetylation of GPX4 is reduced in cadmium (Cd)-induced AKI, thereby aggravating ferroptosis [37]. Additionally, HDAC3-mediated epigenetic modification of the GPX4 promoter suppresses its transcription, further promoting ferroptosis [38]. GPX4 degradation is regulated by WBP2, which competes with heat shock cognate protein 70 (HSC70) to slow chaperone-mediated autophagy, thereby exerting a protective effect during AKI [39]. NRF2 is a well-known transcription factor of GPX4, and recent studies have identified PRDM16 as a novel transcription factor that protects against CLP-induced AKI by targeting the NRF2/GPX4 axis [40].

Mitochondrial dysfunction, ferritinophagy, iron toxicity, transcriptional regulation, and so on are all involved in the regulation of ferroptosis-mediated AKI. Characteristic morphological hallmarks of ferroptosis including mitochondrial contraction, increased membrane density, and loss of cristae [41, 42]. SYVN1, an E3 ubiquitin ligase, promotes ubiquitination and degradation of sulfide:quinone oxidoreductase (SQOR), which in consequence results in mitochondrial dysfunction and ROS accumulation, thereby inducing ferroptosis and renal injury in cisplatin-induced AKI model [43]. STING facilitates NCOA4-dependent ferritinophagy, thereby promoting ferroptosis in ischemic AKI [44]. ACSL4, recognized as a key promoter of ferroptosis, is negatively regulated by HIF-1α in IRI- and FA-induced AKI [45]. Hemopexin accumulation in injured tubular cells results in iron toxicity, ultimately culminating in ferroptosis [46].

#### Cuproptosis

Cuproptosis, triggered by copper (Cu) ion accumulation, contributes to AKI. Excess Cu triggers lipid peroxidation, protein aggregation, and tubular epithelial cell (TEC) damage, thereby impairing cellular function. Cu-associated proteins such as FDX1, DLD, DLAT, DBT, PDHA1, and ATP7A have been implicated in AKI [47]. Cisplatin elevates Cu levels, and Cu supplements exacerbate cisplatin-induced human kidney proximal TEC (HK-2) cell death, mitochondrial dysfunction, and oxidative stress—effects that are partially alleviated by SLC31A1 knockdown, indicating the involvement of Cu deposition in AKI [48].

### Inflammation

Inflammatory damage is pivotal for AKI development. After AKI onset, neutrophils, monocytes/macrophages, dendritic cells, and lymphocytes rapidly infiltrate renal tissue and secrete proinflammatory cytokines (e.g., IL-6, TGF-β, TNF), exacerbating renal injury [49, 50]. These cytokines activate downstream signaling pathways, triggering cascades of inflammation and fibrosis. Renal TECs are the primary targets of AKI and respond by releasing substantial amounts of proinflammatory cytokines, including TNF-α, MCP-1, and IL-8, further amplifying the inflammatory milieu. Most studies have focused on the classical pattern recognition receptors TLR2 and TLR4, which regulate T lymphocyte activation and amplify renal immune responses [51]. Inflammatory injury, triggered by various damaging stimuli, leads to dysfunction and structural compromise of the microvascular endothelial dysfunction within the renal tubulointerstitium.

**Fig. 1:**
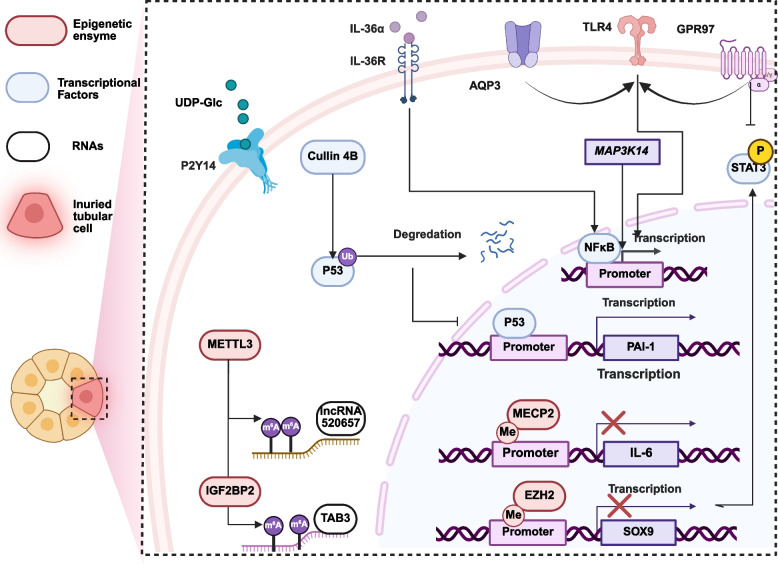
Mechanism of tubular cell inflammation in AKI. Progression of the molecular mechanism of tubular cell inflammation in AKI. GPR97 and AQP3 have been demonstrated to be involved in AKI by influencing the traditional receptor TLR4. The DAMP UDP-Glc, released into the urine, can activate the receptor P2Y14. IL-36R and MAPK14 target the traditional pro-inflammatory transcription factor NF-κB, thus influencing the expression of inflammatory cytokines. Cullin 4B promotes the degradation of P53 through ubiquitination and suppresses the transcription of PAI-1, consequently inhibiting inflammation signaling. METTL3 and IGFBP7 influence the stabilization of RNAs, including lncRNA 520657 and TAB3 mRNA, via m6A modification, promoting downstream inflammation. While MECP2 and EZH2 bind to the methylation site of the promoter region, thus blocking the transcription of IL-6 and SOX9. Created in BioRender. xu, x. (2026) https://BioRender.com/cuii9la

**Fig. 2:**
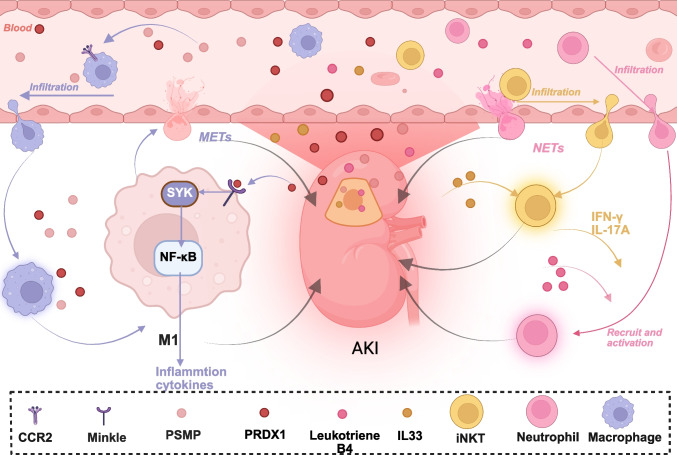
Immune cells in AKI. Novel mechanisms of immune cell contributions to AKI pathogenesis. New findings reveal that the interaction between macrophages and tubular cells, leading to M1 polarization, is associated with the release of PSMP and PRDX1 by damaged tubular epithelial cells. This discovery shows that these DAMPs trigger the expression of inflammatory cytokines, exacerbating damage to tubular epithelial cells. Notably, PRDX1 binds to a specific receptor Mincle and activates the SYK/NF-κB signaling pathway. Furthermore, DAMPs IL-33 and leukotriene B4 are found to recruit iNKT cells and neutrophils, respectively. iNKT cells in the damaged kidney secrete IFN-γ and IL-17A, enhancing neutrophil recruitment and activation. Additionally, NETs and METs produced by macrophages and neutrophils are implicated in the pathogenesis of AKI, working together with iNKT cells and neutrophils. Created in BioRender. xu, x. (2026) https://BioRender.com/kxo6k5s

**Fig. 3:**
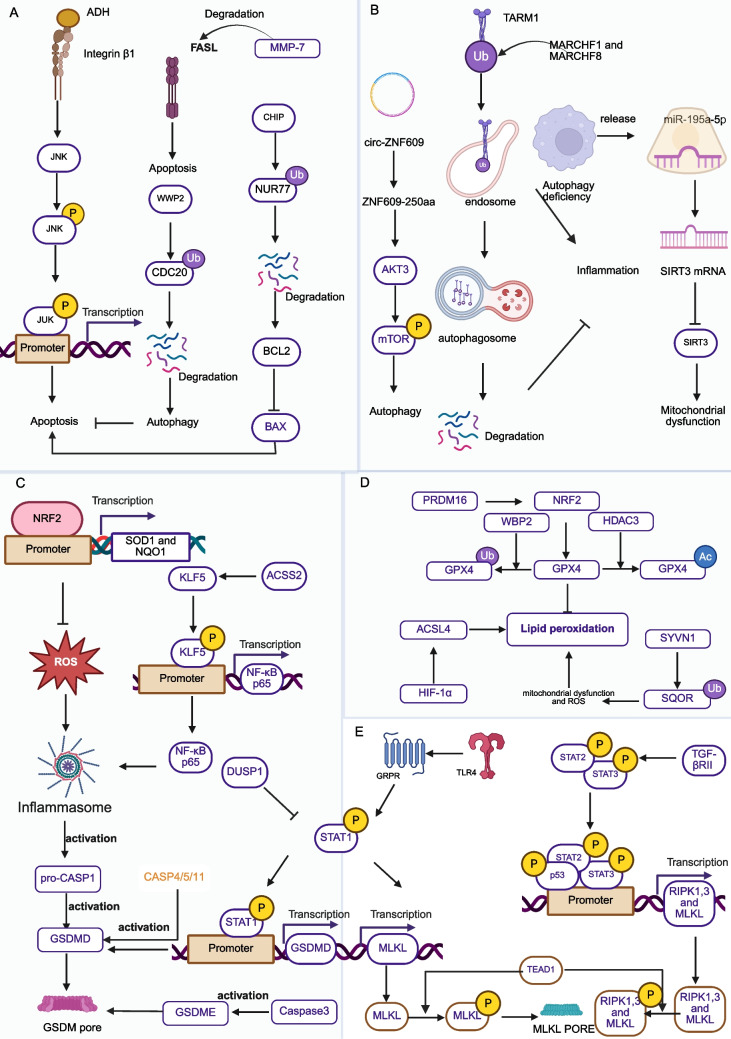
RCD in AKI. **a**. Apoptosis: The signaling pathways leading to apoptosis involve ADH/integrinβ1/JNK, MMP/FASL-FAS, WWP/CDC20/autophagy, and CHIP/NUR77/BCL2 signaling. **b**. Autophagy: Autophagy alleviates TARM1-induced inflammatory damage. Autophagy-deficient macrophages release miR-195a-5p, activating mitochondrial dysfunction in PTE2Cs related to SIRT3 deficiency. The Circ-ZNF609-encoded ZNF609-255aa protein activates AKT3/mTOR/autophagy. **c**. Pyroptosis: It is mediated by the activation of the NRF2/ROS and KLF5/NF-κB inflammasomes. Key steps include caspase activation leading to GSDMD cleavage and pore formation. **d**. Ferroptosis: Key steps in ferroptosis involve lipid peroxidation. PRDM16/NRF2/GPX4 play an anti-lipid peroxidation role, while HDAC3 and WBP2 dampen the anti-lipid peroxidation role of GPX4 through epigenetic modification. HIF-1α/ACSL4 and SYVN1/SQOR ubiquitination also promote peroxidation and ferroptosis. **e**. Necroptosis: The necroptotic pathway is mediated by TLR4/GRPR signaling, leading to STAT1 transcriptional increase in the expression of MLKL. While TGF-βRII/STAT2 signaling promotes the expression of all these necroptosis markers, RIPK1, RIPK3, and MLKL. TEAD1 promotes the phosphorylation of key elements RIPK1, RIPK3, and MLKL, resulting in MLKL pore formation and cell death. Created in BioRender. xu, x. (2026) https://BioRender.com/6l5tp9x

Recent studies have uncovered novel molecular mechanisms underlying TEC inflammation (Fig. 1), macrophage polarization, Treg–neutrophil interactions, and inflammatory signaling pathways during AKI (Figs. 2 and 3).

#### TECs inflammation during AKI

TEC inflammation is triggered by IR, nephrotoxic agents, ROS, lipopolysaccharide (LPS), and DAMPs.

Fundamentally, the transcription factor p53 plays a pivotal role in the pathogenesis of AKI. Plasminogen activator inhibitor-1 (PAI-1), which is typically expressed in endothelial cells, is elevated in damaged TECs under pathological conditions. Studies have demonstrated that Cullin 4B (CUL4B) deletion exacerbates kidney injury and inflammation via the p53/PAI-1 axis in mouse models of cisplatin- and IR-induced AKI [52]. This exacerbation is mediated by reduced CUL4B—dependent ubiquitination, which leads to subsequent degradation of p53 and promotes downstream inflammatory responses. In cisplatin- and folate-induced AKI, a noncanonical NF-κB activation pathway involving mitogen-activated protein kinase kinase kinase 14 (MAP3K14) has been identified. MAP3K14 induces NF-κB2 p100/52 expression, reducing macrophage infiltration and proinflammatory cytokine production [53]. During the transition from AKI to CKD, EZH2 promotes M2 macrophage polarization by activating the STAT6 and PI3K/AKT signaling pathways [54]. EZH2 also contributes to tubular senescence and chronic injury via interaction with the aryl hydrocarbon receptor (AhR) [55]. The transcription factor FOS-like 1 (Fosl1) exerts a protective effect in cisplatin-induced AKI by targeting the Klotho promoter [56].

Epigenetically, methyltransferase-like 3 (METTL3) binds to hsa-lncRNA 520657 and TAB3 mRNA [57–59], promoting AKI progression through N6-methyladenosine(m⁶A) modification. While interacting with IGF2BP2, METTL3-related m6A modification stabilizes TAB3 mRNA, thereby promoting cisplatin- and LPS-induced AKI [59]. Methyl-CpG-binding protein 2 (MeCP2) binds to the IL-6 promoter, suppressing IL-6 expression and downregulating STAT3 phosphorylation. MeCP2 knockdown activates this pathway, inducing inflammation and apoptosis in IR AKI [60]. Histone methyltransferase enhancer of zeste homolog 2 (EZH2) participates in multiple phases of AKI by modulating distinct signaling pathways. In septic AKI, EZH2 binds to the SOX9 promoter and inhibits its transcription via methylation, influencing inflammation and apoptosis through the Wnt/β-catenin pathway [61].

Specifically, GPR97, a newly identified GPCR, promotes ischemia- and toxin-induced inflammation via activation of Sema3A and TLR4 signaling [62]. IL-36α, a novel member of the IL-1 family, binds to the IL-36 receptor on proximal tubular cells, activating canonical NF-κB signaling and promoting ERK phosphorylation [63]. In sepsis-induced AKI, fortunellin mitigates renal injury by reducing inflammation via the TLR4/NF-κB pathway [64]. Aquaporins modulate inflammatory signaling in AKI pathogenesis: AQP3 regulates inflammation through TLR4 signaling [65], while AQP1 modulates inflammation via the PI3K pathway [66]. The NLRP3 inflammasome promotes maturation of IL-1β and IL-18, exacerbating AKI induced by cisplatin, IR, sepsis, and other insults [67–69]. Additionally, G protein-coupled receptors (GPCRs), including D1-like (D1, D5) and D2-like (D2-D4) subtypes—play roles in this complex inflammatory network [70].

Damaged TECs recruit immune cells, such as macrophages and neutrophils. Interestingly, DAMPs are secreted into urine; for example, UDP-glucose is highly concentrated in the urine of patients with cardiac surgery–induced AKI. It binds to the P2Y14 receptor on proximal tubular cells, inducing cytokine release and promoting immune cell recruitment [71].

#### Macrophage polarization and Treg/neutrophil interactions in the pathogenesis of AKI

Macrophage subsets, including M0, M1, and M2 macrophages, plays different role in the pathogenesis of AKI. M1 and M2 macrophages differentiate from M0 precursors. M1 macrophages exacerbate inflammation by secreting proinflammatory cytokines, chemokines, and ROS, whereas M2 macrophages contribute to tissue repair and exert immunosuppressive effects.

**Fig. 4:**
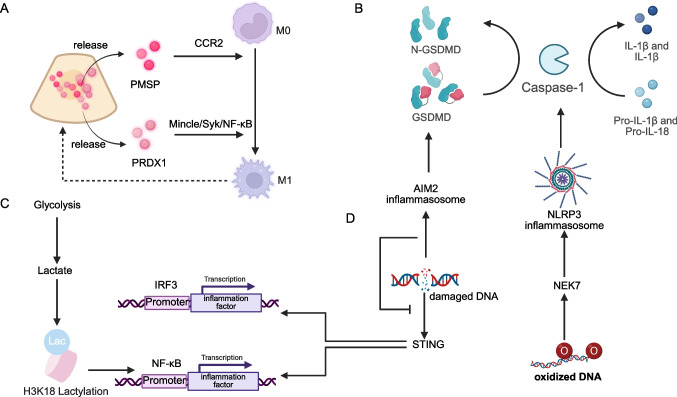
Crosstalk among cellular mechanisms in AKI. **a**. Macrophages and PTECs: Injured PTECs release PMSP and PRDX1, which separately promote the differentiation of macrophages into the M1 phenotype through CCR2 and the Mincle/Syk/NF-κB pathway, thereby modulating inflammatory responses and exacerbating inflammation-induced injury to the PTECs. **b**. Pyroptosis and Inflammation: The activation of inflammasomes such as AIM2 and NLRP3 leads to the cleavage of GSDMD by caspase-1, resulting in the formation of plasma membrane pores and release of IL-1βand IL-18. This process, known as pyroptosis, amplifies inflammation by promoting the secretion of pro-inflammatory cytokines like IL-1βand IL-18 are released. **c**. Metabolism and Inflammation: Metabolic shifts, including enhanced glycolysis and elevated lactate production, exert an influence on inflammatory responses. Lactate is capable of inducing H3K18 lactylation, which subsequently modulates the transcription of inflammation—related genes mediated by NF—κB. **d**. DNA Damage, inflammation, and pyroptosis: DNA damage triggers the activation of the AIM2 inflammasome and the STING pathway. The activation of the AIM2 inflammasome by damaged DNA, which leads to pyroptosis, can exert an inhibitory influence on the inflammation response related to STING signaling. Created in BioRender. xu, x. (2026) https://BioRender.com/6bn14d9

Single-cell sequencing has revolutionized our understanding of AKI by unveiling macrophages as central orchestrators of immune responses, with their dynamic spatiotemporal roles spanning all stages of injury progression. Spatial transcriptomic analysis of renal tissues at 1, 3, 14, and 28 days post-IRI demonstrated that macrophages, particularly pro-inflammatory and pro-repair subsets, exhibit distinct migration dynamics and functional polarization [72]. Interestingly, the S3 segment of renal tubules was identified as the primary site of early macrophage infiltration, preceding neutrophil recruitment. Furthermore, macrophages were found to work in concert with neutrophils to precisely regulate the inflammatory response, highlighting the complex and coordinated nature of the immune response in AKI. Heterogeneity among IRI-induced macrophages is striking, with Ly6ChiF4/80lo infiltrating macrophages (IMs) driving early inflammation via CCR2-mediated signaling and transcriptional programs regulated by NF-κB1, Jun, and Cebpb [73] (Fig. 4). Moreover, Single-cell sequencing revealed TIGIT's key regulatory role in AKI inflammation, opening new avenues for targeted inmmune-modulating therapies [74]. Furthermore,in 2022, Klocke et al.'s study via single-cell RNA sequencing detected injury-related TEC changes in AKI patients, indicating damage severity and aiding in finding biomarkers and therapeutic targets [75]. While polyploid tubular cells play a dual role in AKI pathogenesis and its progression to AKI-CKD [76].

Further, a study integrating spatial transcriptomics, single-cell sequencing, and CODEX immunofluorescence revealed key renal epithelial-immune cell interactions across AKI mouse models. In the IRI model, neutrophils in the outer medulla accounted for 52.2% of all infiltrating neutrophils, with the proximal tubule S3 segment showing high colocalization with neutrophils. This interaction is regulated through elevated ATF3 expression, which modulates neutrophil migration. In the CLP model, infiltrating macrophages in the outer cortex represented 80.2% of the total. Crucially, cortical proximal tubule cells engage in cross-talk with macrophages may via high expression of Midkine, directly participating in macrophage recruitment and activation processes [77]. However, the underlying cross-talk mechanisms of activated immune cells and injured tubular cells require further exploration.

In addition to single-cell sequencing, a series of molecules involved in macrophage-related AKI have also been discovered. Upon toxic or ischemic injury, proximal tubular cells release peroxiredoxin 1 (PRDX1), which promotes M1 polarization and activates the Mincle/Syk/NF-κB pathway, increasing the expression of IL-1β, IL-6, and TNF-α [78] (Fig. 4). Macrophage extracellular trap (METs) formation exacerbates AKI following glycerol injection via Mac-1 expression, which also facilitates interaction with platelets [79].

The mechanisms by which diverse types of T cells are involved in AKI have been comprehensively elucidated. Cytokines such as IL-17A, IL-2, and IL-33 (encoded by IL233) alleviate AKI by targeting regulatory T cells (Tregs) [80, 81]. MeRIP-seq and RNA-seq identify the crucial role of ALKBH5, genetic inhibition of ALKBH5 prevents the stabilization of CCL28 mRNA via m6A modification, thereby enhancing Treg recruitment and ameliorating kidney inflammation in IR AKI [82]. IR-injured kidneys recruit invariant natural killer T (iNKT) cells, which produce IFN-γ and IL-17A, sustaining neutrophil infiltration and activation at the injury site [83]. NRF2 knockout mice are highly susceptible to renal injury from IR and cisplatin [84], whereas conditional Keap1 knockout in T cells confers renal protection [85].

Other immune cells and mediators also contribute to the progression of AKI. Leukotriene B_4_ and its receptor mediate neutrophil recruitment during cisplatin-induced AKI [86]. Neutrophil extracellular trap (NET) components are elevated in patients with AKI following cardiac surgery, with DNA-binding protein YB-1 as a key modulator [87]. Deficiency of the E-prostanoid 3 (EP3) receptor in myeloid cells blocks TLR4-mediated inflammatory signaling, thereby reducing tubular cell apoptosis and necroptosis, ultimately ameliorating AKI [88].

### DNA damage

DNA damage is a continuous biological process occurring under both physiological and pathological conditions, with a substantial proportion of lesions that can be repaired. Damage to both mtDNA and nDNA is well established as a contributor to AKI pathogenesis, particularly in cisplatin-induced AKI. Inadequate DNA repair can exacerbate inflammatory responses and accelerate cellular senescence [89]. Cyclin-dependent kinase 12 (CDK12) contributes to genome stabilization during cisplatin-induced AKI [90]. DNA repair comprises multiple coordinated steps, including damage site recognition, excision, homologous recombination, and ligation. Key proteins, such as PIKK, ATM, and DNA-PKcs, orchestrate these processes and significantly influence AKI outcomes [91].

Additionally, damaged DNA can activate the CGAS-STING signaling pathway, thereby aggravating AKI and promoting its progression to AKI-CKD [89]. This pathway, which is currently a focal point of research, can be triggered by both endogenous and exogenous DNA. Under physiological conditions, nuclear and mitochondrial DNA are spatially restricted and do not activate CGAS because of steric hindrance [92, 93]. However, when mitochondrial DNA (mtDNA) is released into the cytoplasm, it is recognized by cGAS, initiating downstream signaling cascades that induce inflammatory responses thus contribute to AKI development. Activation of the cGAS-STING pathway by mtDNA release also involves BAX phosphorylation, which is associated with increased levels of phosphoglycerate mutase 5 (PGAM5) in IR-induced AKI [94]. Protective interventions, such as microRNA-155 (miR-155) [95], nicotinamide mononucleotide (NMN) [96], and vitamin D [97] have demonstrated efficacy in mitigating DNA damage and attenuating AKI severity.

Different mechanisms are involved in mediating DNA damage, pyroptosis, and inflammation under different stimuli. A new study published in 2025, which focused on oxidized ds-DNA, found that oxidized ds-DNA activates the NEK7/NLRP3 signaling pathway, thereby stimulating macrophage pyroptosis and promotes the development of cisplatin-induced AKI [98]. Meanwhile, pyroptosis induced by dsDNA in macrophages, which is recognized by AIM2, triggers rapid pyroptotic responses. These responses dampen the inflammatory response, thereby ameliorating rhabdomyolysis—induced AKI [99] (Fig. 4).

### Metabolism disorder

The kidney is a highly metabolic organ, and its proximal TECs have exceptionally high energy demands.

TECs primarily rely on fatty acid metabolism, with key enzymes including CPT1, ACADL (LCAD), and medium-chain acyl-CoA dehydrogenase (MCAD). When cisplatin induces AKI, the expression of these enzymes declines, leading to reduced fatty acid oxidation and adenosine triphosphate (ATP) production. Epigenetic modifications regulate the functions of these enzymes. SIRT3 regulates fatty acid metabolism via its deacetylase activity and exerts a protective effect on AKI [100]. Reduced ATP production impairs cytoskeletal assembly, resulting in the depletion of brush borders and detachment of TECs. Additionally, impaired fatty acid metabolism promotes lipid droplet accumulation, triggering lipotoxicity and inflammatory responses that exacerbate AKI. Lipids such as palmitic acid (PA) can trigger both ferroptosis and ER stress [101, 102]. However, trimetazidine, CYP enzymes, and FXR offer hope for AKI treatment. Trimetazidine mitigates lipid-induced inflammation, whereas CYP enzymes and FXR promote lipid catabolism and reduce lipogenesis [103]. The tricarboxylic acid (TCA) cycle is central to cellular energy metabolism. During mitochondrial electron transport, electron leakage can generate ROS [104, 105], which induce oxidative stress and cellular damage. Manganese superoxide dismutase (MnSOD), located in the mitochondrial matrix, and Cu/zinc SOD, present at low concentrations in the intermembrane space provide antioxidant defense [105, 106]. Hydrogen peroxide (H_2_O_2_) that escapes the mitochondria is detoxified by the glutathione peroxidase and thioredoxin/peroxiredoxin systems to maintain redox homeostasis. Lysosomes also contribute to ROS clearance, further supporting cellular integrity [107].

Recent studies utilizing omics technologies have uncovered the impact of diverse metabolic processes on AKI pathogenesis and their underlying molecular mechanisms. The thick ascending limb (TAL), the kidney's most metabolically active region, relies on oxidative phosphorylation and fatty acid oxidation for ATP production. Single-cell sequencing shows that propofol, a clinical sedative, impairs TAL oxidative phosphorylation, shifting metabolism to glycolysis and worsening tubular damage in mouse IRI-AKI models [108]. Multi-omics integration analysis uncovers the core mechanisms of ferroptosis-lipid metabolism crosstalk, emphasizing significant associations among iron metabolism, lipid metabolism, and ferroptosis in renal tubular epithelial cells. It identifies direct correlations between abnormal iron metabolism and elevated ferroptosis levels, with key molecules FTL/FTH1 (iron metabolism) and GPX4 (lipid metabolism) showing high co-expression, regulated by KLF6 via transcriptional inhibition of these factors [109]. Another study focusing on cholesterol metabolism, through integrated analysis of single-cell spatial transcriptomics, metabolomics, and scRNA-seq, discovered that the cholesterol metabolite 27-Hydroxycholesterol specifically accumulates in damaged renal tubular regions [110]. It activates the estrogen receptor α endogenous ligand via receptor-ligand mode and upregulates downstream HMOX1 expression, ultimately triggering lipid peroxidation cascades to promote ferroptosis [110].

PTCs undergo pronounced metabolic reprogramming, transitioning from oxidative phosphorylation to glycolysis. This metabolic shift enables a more rapid acquisition of energy but concurrently results in the accumulation of lactate [111]. The increased lactate modulates the progression of AKI through epigenetic modifications. Specifically, the lactylation of Aldehyde Dehydrogenase 2(ALDH2) influences the ubiquitin-mediated proteasomal degradation of the mitophagy receptor PHB2 via protein–protein interactions. This, in turn, leads to mitochondrial dysfunction, ultimately contributing to the pathogenesis of AKI [112]. In S-AKI, The lactylation modification of histone H3 lysine 18 lactylation (H3K18la) activates the promoters of the Ras homolog gene family member A and NF-κB, thereby promoting inflammatory responses, cellular apoptosis, and other pathological processes [113] (Fig. 4).

Arginase 2 is an enzyme involved in arginine metabolism in the kidney, which is upregulated in the renal tubular mitochondria of mice and in the serum of patients with contrast-induced AKI (CI-AKI). Its inhibition mitigates renal dysfunction, nitrosative stress, and tubular apoptosis, effects also observed in cisplatin- and vancomycin-induced AKI. Mechanistically, ARG2 promotes these effects in part by suppressing HO-1, with CREB1 identified as a potential transcription factor. Proteomic and transcriptomic analyses confirm ARG2 accumulation across multiple drug-induced AKI models [114].

20-HETE serves as a metabolite derived from arachidonic acid (AA). Deficiency of 20-hydroxyeicosatetraenoic acid (20-HETE) exacerbates ischemic AKI in Dahl salt-sensitive (SS) rats, leading to elevated creatinine levels and medullary hypoperfusion. In contrast, CYP4A gene transfer from Brown Norway (BN/Lew) rats restores 20-HETE levels and confers renal protection. This finding reveals an SS-specific mechanism: genetic loss of 20-HETE disrupts medullary blood flow regulation, increasing susceptibility to ischemic injury, while CYP4A-mediated 20-HETE replenishment restores medullary perfusion and mitigates ischemic injury [115].

## Innovative diagnostic approaches

The emergence and clinical deployment of diagnostic biomarkers and innovative tools have ushered in a transformative era in AKI research. In recent years, early biomarkers for AKI, notably urinary TIMP-2, IGFBP7 [116], NGAL [117], KIM-1 [118], and non-coding RNAs, have been extensively evaluated and applied, aiding in early detection, differential diagnosis, and prognostic assessment. Notably, combined urinary levels of TIMP-2 and IGFBP7 can effectively predict the onset of AKI stages 2 to 3 and currently represent the only AKI biomarker approved by the U.S. Food and Drug Administration (FDA) for clinical applications. As an independent predictor, NGAL can accurately forecast AKI prognosis based on its concentration in both blood and urine. The discovery of non-coding RNAs, including lncRNA [57, 119, 120], miRNA [121, 122], and circRNA [15, 123], offers novel biomarker alternatives for the early diagnosis of septic AKI, often surpassing traditional markers in diagnostic sensitivity. The glomerular filtration rate (GFR) is indispensable for assessing renal function, and its dynamic monitoring is pivotal in kidney disease research and diagnosis. Although real-time clinical GFR monitoring technologies are not yet widely available, recent studies have introduced skin-based measurement techniques using fluorescent compounds such as MB-102 [124, 125], enabling real-time GFR tracking [126]. The diagnostic utility of biomarkers varies across clinical contexts, including early detection, severity grading, and prognostic evaluation. NGAL serves as a marker of distal tubular injury, while elevated KIM-1 levels indicate medullary hypoxic damage [127]. Soluble Toll-like receptor 2 (sTLR2) serves as a biomarker for sepsis-induced AKI [127], and IL-6 can predict AKI both in septic and post-cardiac surgery settings [128]. KNP-1, a kidney-targeting fluorescent probe activated by the early AKI biomarker peroxynitrite (ONOO⁻), enables detection at least 24 h earlier than conventional methods, offering high specificity and facilitating timely medical intervention [6].

In the future, the diagnostic paradigm for kidney diseases is expected to increasingly incorporate AI, with the improvement of diagnostic technology. Photoresponsive nanomaterials [129], Shear wave elastography (SWE) with contrast-enhanced ultrasound (CEUS) are novel techniques for AKI diagnosis. Among these, CEUS and SWE play a significant role in identifying and evaluating renal hemodynamics [130]. The integration of virtual learning collaborative frameworks with automated dashboard systems constitutes a highly promising management paradigm for contrast-associated AKI prevention, demonstrating a statistically significant 40% reduction in AKI risk (adjusted OR = 0.60, *p* < 0.05) compared to technical assistance alone [131]. AI models are capable of predicting the occurrence of AKI before significant changes in biochemical indicators by analyzing clinical data. A nationwide multicenter study developed a deep learning model that dynamically predicts the in-hospital risk of AKI based on data from 7,084,339 hospitalized patients, providing support for early intervention [132]. Meanwhile, machine learning (ML) models have achieved accurate AKI risk classification in some clinical settings, but their real-world performance still requires further validation [133]. However, this evolving diagnostic paradigm faces several challenges. These include assessing the prognostic impact of automated urine volume monitoring systems, balancing the safety and efficacy of high-dose furosemide, evaluating the diagnostic utility of automated urine sediment microscopy, and optimizing dynamic AKI assessment strategies [134]. Addressing these challenges will be pivotal in shaping future research directions in the diagnosis and management of kidney disease.

## Groundbreaking advancement in treatment

Currently, the treatment of AKI primarily focuses on symptomatic and supportive care, with no universally effective drug currently available to directly target AKI. However, numerous promising pharmacological agents and compounds are undergoing clinical trials. Through bioinformatics analysis, several traditional drugs with potential therapeutic value have been identified to exert protective effects in AKI, including reduced glutathione, vitamin D [135, 136], dexmedetomidine [137], α-lipoic acid [138], fortunellin [64], and formononetin.

Researchers have developed several anti—inflammatory pharmacological agents in animal models of AKI in the recent decade. By investigating key molecular targets and employing techniques such as molecular docking of small-molecule compounds, researchers have discovered several therapeutically significant compounds. For example, SKLB023 targets TLR4 signaling, alleviating pathological damage, inflammation, and apoptosis in septic AKI [139].

Nanomaterials exert anti-inflammatory actions via their intrinsic material characteristics as well as through the loading of molecules, in addition to other approaches. CF@PDA, a nanomaterial composed of curcumin and Fe^3⁺^, exhibits SOD and catalase activities. It effectively targets and eliminates ROS, thereby protecting human proximal tubular cells and the kidneys from injury. Additionally, it inhibits the ROS–inflammation cascade by reducing macrophage polarization from the M0 to M1 phenotype [140]. IL-10 is an anti-inflammatory cytokine; however, its clinical application is limited by a short half-life and off-target effects. However, administration of IL-10-loaded extracellular vesicles (EVs) every 24 h for three days following unilateral IRI has shown therapeutic promise in AKI by promoting M2 macrophage polarization [141]. The PSMP antibody also targets macrophages to alleviate inflammation in IR-induced AKI [73]. Se@BSA promotes the expression of GPX-1, thereby mitigating NLRP3/caspase-1–mediated kidney inflammation [142].

Targeting RCD is also involved in AKI therapeutic research. Among various types of RCD, ferroptosis has played a pivotal role in research over the past decade. Inhibiting ferroptosis can mitigate its detrimental effects [143]. Silibinin inhibits ferroptosis by disrupting the interaction between NCOA4 and FTH1 [144]. Notably, FTH1 knockdown in vitro reversed the suppressive effect of silibinin on ferroptosis. Additionally, compounds such as tiliroside, loureirin C, dexmedetomidine, and andrographolide inhibit ferroptosis via activation of the NRF2 pathway, thereby demonstrating protective effects against AKI [145–147]. Traditional drugs such as 7-hydroxycoumarin ameliorate AKI by inhibiting RIPK1/RIPK3-mediated necroptosis and promoting SOX9/cyclin D1-dependent proliferation. However, the molecular mechanisms underlying the interaction between 7-hydroxycoumarin and its downstream targets remain incompletely elucidated [148].

Other therapetic regents, mesenchymal stem cells (MSCs) facilitate renal repair by modulating the balance of innate immunity. Emodin, a traditional Chinese medicine constituent, may alleviate IRI induced AKI through anti-apoptotic and anti-inflammatory mechanisms [149].

In the clinical setting, numerous promising pharmacologic agents and compounds are currently undergoing clinical trials. However, recombinant alkaline phosphatase [150], vitamin D [151], and other drugs have not demonstrated positive results. Effective volume management is paramount for the symptomatic and supportive treatment of AKI. Excessive volume can precipitate or worsen heart failure, whereas insufficient volume can lead to shock and exacerbate AKI. In response to this clinical challenge, the Acute Disease Quality Initiative(ADQI) introduced the concept of a continuous renal replacement therapy (CRRT) feedback system in 2016, advocating for dynamic and autonomous AI-based regulation [152]. By 2022, the 27th ADQI conference had aimed to develop a framework for the proper development, validation, and implementation of digital health in AKI care (DHAKI). The conference proposed a management model that encompassed diagnosis, prevention, and treatment across multiple levels, spanning households, communities, regions, and nations. This model represents a forward—looking paradigm in AKI management [153].

## Limitation

Most AKI therapeutic strategies derive from in vitro and preclinical models, facing substantial clinical translation challenges. Preclinical models often fail to recapitulate the complexity of human AKI, leading to overestimated therapeutic efficacy in preclinical studies. A prominent translational gap exists, with promising agents showing no clinical benefit. Novel therapies also confront hurdles like inconsistent efficacy, unstandardized dosages, long-term safety concerns, and lack of etiology-specific stratification.

## Conclusion

Over the past decade, AKI research has achieved pivotal advancements, including the concept definition update from ARF to AKI and the proposed AKD framework emphasizing early CKD prevention, alongside refined mechanistic insights into regulated cell death, inflammation, and metabolic disorders. Diagnostic progress encompasses urinary biomarkers, non-coding RNAs, and AI-assisted models, while emerging therapies complement conventional supportive care. Future research should center on DHAKI framework advancement for AI-enabled monitoring, and targeted therapy translation via etiology-classified trials, aiming to enhance renal repair and improve outcomes.

## Data Availability

This review article does not report original research data. All data referenced in thismanuscriptReferencesare derived from published studies and areavailable in the original publications cited in the section.
